# Gold nanoparticles decorated biguanidine modified mesoporous silica KIT-5 as recoverable heterogeneous catalyst for the reductive degradation of environmental contaminants

**DOI:** 10.1038/s41598-021-82242-z

**Published:** 2021-02-02

**Authors:** Hojat Veisi, Parasto Abassi, Pourya Mohammadi, Taiebeh Tamoradi, Bikash Karmakar

**Affiliations:** 1 Department of Chemistry, Payame Noor University, 19395-4697 Tehran, Iran; 2 Department of Chemistry, Gobardanga Hindu College, 24-Parganas (North), India

**Keywords:** Catalysis, Heterogeneous catalysis, Materials science, Nanoscience and technology

## Abstract

This current study involves the novel synthesis of Au nanoparticles (Au NPs) decorated biguanidine modified mesoporous silica KIT-5 following post-functionalization approach. The tiny Au NPs were being stabilized over the in situ prepared biguanidine ligand. The high surface area material was characterized using analytical techniques like Fourier Transformed infrared (FT-IR) spectroscopy, N_2_-adsorption–desorption isotherm, Scanning Electron Microscopy (SEM), Transmission Electron Microscopy (TEM), Energy Dispersive X-ray Spectroscopy (EDS), and X-ray Diffraction study (XRD). Our material was found to be an efficient catalyst in the reductive degradation of harmful water contaminating organic dyes like Methylene blue (MB), Methyl Orange (MO) and Rhodamin B (RhB) in presence of NaBH_4_ at room temperature. The whole procedure was followed up with the help of time dependant UV–Vis spectroscopy. All the reactions followed pseudo-unimolecular kinetics and corresponding rate constant were determined. The reduction rate becomes high in presence of higher load of catalysts.

## Introduction

Throughout the last few decades, with the population boom, sophistication in socialism and industrial development has come into prominence as natural demand. However, lack in consciousness and inadequate control measures has in turn increased the pollution and exhaustion in environmental stability. The release of harmful contaminants into air and water are causing serious threat to human ecology^1–3^. Many of the contaminants, such as, drugs and pharmaceuticals, hormones, dyes, personal care products (PCPs), endocrine-disrupting chemicals (EDCs) and other recalcitrant organic compounds are chronically toxic, unrelenting, hardly decomposed and mostly water soluble^4–6^. Contagion of these substrates into wastewater effluent and thereafter into natural waters is a major issue in current days^7–9^. A bulk amount of synthetic dyes is released into water by textile industries as a result of incomplete quenching of pigments and successive washing of colored materials^10,11^. Even if at feeble concentrations, dyes are highly diffusible into water which impedes the penetration of sunlight. This reduces the dissolution of oxygen into water, causes death of photosynthetic organisms which ultimately leads to disruption of aquatic ecosystem^12^. Moreover, the organic dyes promotes the photocatalytic oxidation of water and increases the concentration of hydroxyl radicals leading to severe rancidity. Hence, wastewater treatment by degradation of these dyes following the development of suitable techniques has been a vital concern in view of sustainable management^13,14^.

**Scheme 1:**
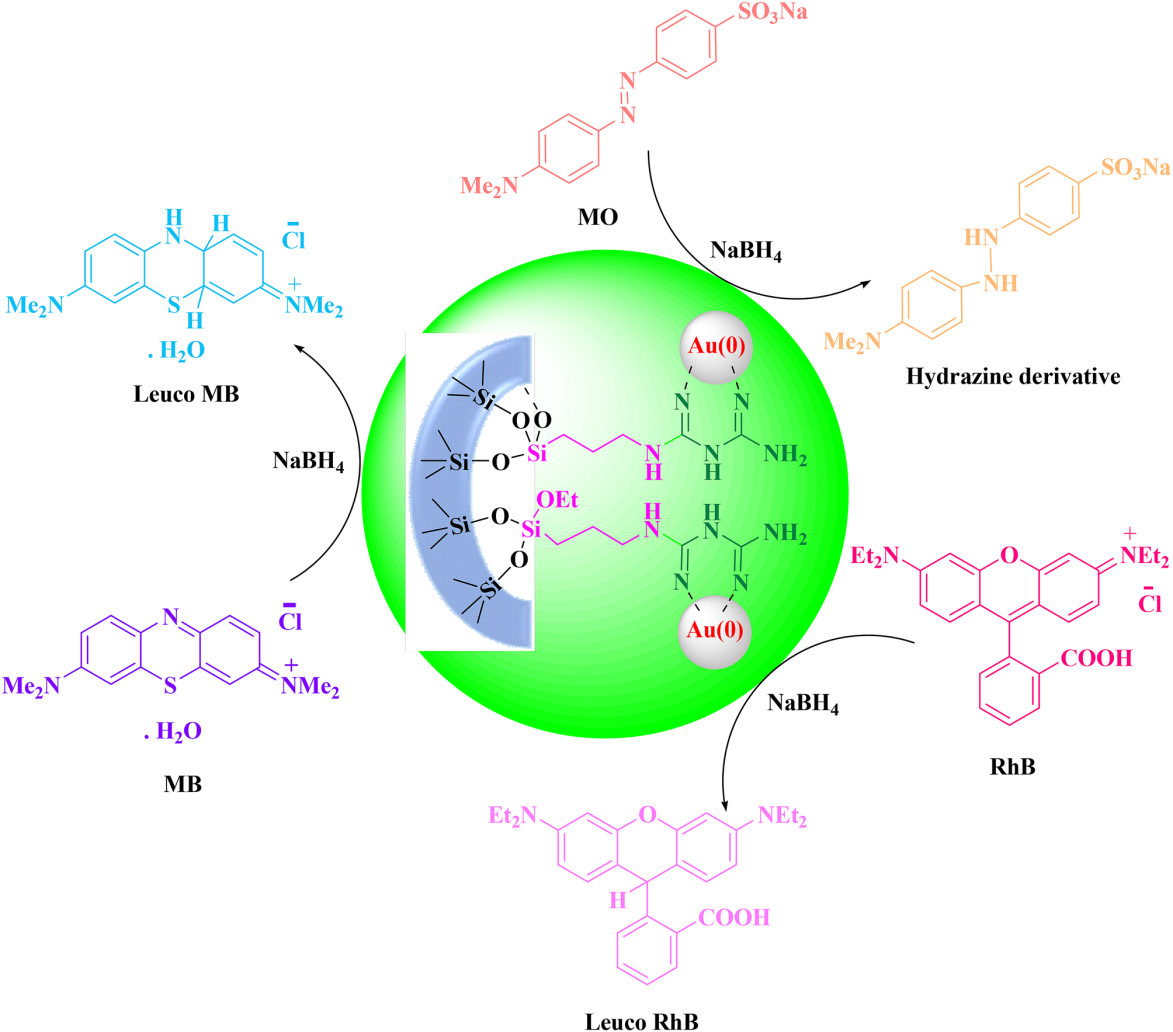
KIT-5-biguanidine-Au(0) catalyzed reduction of organic dyes.

Among the different physical and chemical methods, adsorption, membrane filtration, photo-degradation, coagulation, chemical and electro-oxidation techniques have been the most effective towards the removal of dyes from aqueous solutions, based on their efficiency and simplicity^15–19^. However, unchanged chemical nature of the contaminants even after the treatment, expensive filters and apparatus, use of harsh chemicals, production of large amount of sludge materials, use of prolonged time irradiation of UV source, requirement of external power sources are the major drawbacks of these techniques^7,20^. Catalytic reduction of dyestuffs involving nanomaterials has been one of the emerging cost-effective and energy efficient green methods in this regard which meets the global requirements. The procedure successfully transforms the organic pollutants to safer chemicals being tender to natural waters. There have been several reports published in recent years involving different kind of nanomaterials to catalyze the reduction of organic dyes^21–29^. Being a part of this study, towards the continuous development of this protocol^27^, we wish to report gold nanoparticle embedded biguanidine functionalized and amine modified mesoporous KIT-5 as a novel nanocomposite catalyst in the reduction of some specific organic dyes, namely, Methylene Blue (MB), Methyl Orange (MO) and Rhodamine B (RhB) using NaBH_4_ as reducing agent (Scheme 1).

Metal nanoparticles (MNP) have accomplished utmost attention to researchers due to their exceptional physical, chemical and biological properties. The nanometric size, high surface to volume ratio, size and shape dependant properties induces special characteristics that leads to extensive applications of MNPs in electronics, optics, bio-sensing, plasmonics, energy conversion, biomedical applications and catalysis^30–32^. Particularly in catalysis, the noble MNPs (Au, Ag, Pd and Pt) are of special interest due to their unique behaviors^33–46^. Nevertheless, due to ultrasmall size, self-aggregation is a common phenomenon that reduces the catalytic activity. Anchoring them over suitable support decrease the tendency to a great extent^47^. Among the different supports, ordered mesoporous silica (OMS) occupy significant position for the past three decades due to their large surface area, tunable pore diameter and pore thickness with uniform distribution. In addition, they could easily be surface functionalized exploiting the large number of surface hydroxyl groups^48–50^. KIT-5 is an interesting three dimensional face centered cage type cubic mesoporous silica with interconnected pore channels^51,52^.

**Scheme 2:**
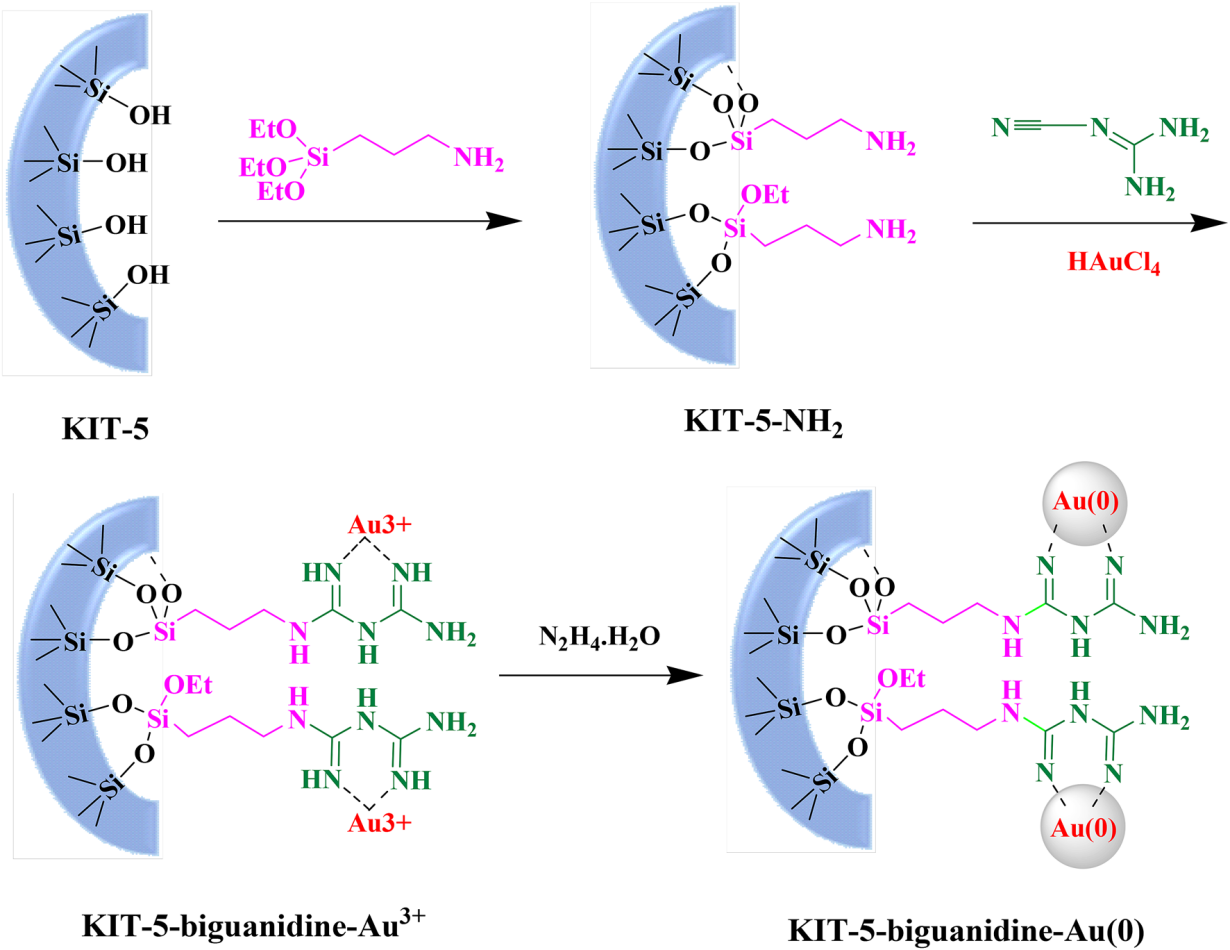
Post grafting synthesis of KIT-5-biguanidine-Au(0) catalyst.

We have utilized KIT-5 as base matrix in our study and decorated its surface first with aminopropyl silica followed by covalent linkage with cyanoguanidine. The resulting biguanidine function acts as an excellent ligand to anchor Au(III) ions as well as a very good stabilizer of Au NPs. The as synthesized nanocomposite (Scheme 2) was proved to be an outstanding catalyst while studying its activity in the reduction of organic dyes. The whole analysis was monitored through UV–Vis spectroscopy.

## Experimental

### Preparation of KIT-5

The mesoporous matrix KIT-5 was prepared following our previously reported method^53^. 19.0 g of the triblock copolymer (Pluronic F127) was mixed to 8.5 g HCl (37%) and 192 g H_2_O and the mixture was heated at 45 °C for 4 h. Then 19.0 g TEOS was added to it and stirred at same condition for overnight followed by hydrothermal aging at 100 °C for 24 h. The resulting white precipitate was filtered carefully and washed sufficiently with distillated H_2_O and dried in air. Finally, the template was removed by calcination at 550 °C for 5 h in air at a ramping rate of 2 °C/min.

### Preparation of KIT-5-biguanidine

1.0 g of KIT-5 was dispersed in 100 mL anhydrous toluene flask and stirred at ambient temperature for 1 h. 1.0 mL 3-aminoropropyl trimethoxysilane (APTMS) was then added dropwise to the reaction mixture and refluxed for overnight under N_2_ atmosphere. After completion, the precipitate was filtered and washed with ethanol (70%) for several times. It was dried subsequently at 60 °C for overnight affording the tripropylamino derivative (KIT-5-NH_2_). In the next step, 0.5 g of the KIT-5-NH_2_ product was added to 100 mL dry acetonitrile and stirred at 40 °C for 30 min. 3.0 mmol of cyanoguanidine was then added to the reaction mixture and heated at 60 °C for overnight. Finally, the as obtained precipitate was filtered, washed with ethanol (70%) for several times and dried at 60 °C for overnight resulting the KIT-5-biguanidine product.

### Preparation of KIT-5-biguanidine-Au(0) nanocomposite

0.5 g of the KIT-5-biguanidine composite was adhered into 50 mL acetonitrile and vigorously stirred at 50 °C for 1 h. A 50 mL acetonitrile solution containing 20 mg of HAuCl_4_ was added to the previous suspension very slowly dropwise and subsequently refluxed for 5 h till the formation of Au(III) complex. Au (0) NP formation was started in situ when hydrazine hydrate (300 µL) was added to it dropwise, as indicated by the change in color to brown–red. The resulting mixture was refluxed for 24 h and finally the grey precipitate was filtered, washed several times with 70% EtOH and dried at 50 °C for overnight in air.

### Catalytic reduction of the organic dyes (OD) over KIT-5-biguanidine-Au (0) nanocomposite

Typically, the nanocatalyst (2–3 mg) was added to a very dilute aqueous solution (3.1 × 10^–5^ M) of OD (10 mL). Then, 2 mL of freshly prepared aqueous NaBH_4_ solution (5.3 × 10^–3^ M) was added to it and the mixture was allowed to stir at room temperature. Progress of the reaction was monitored by recording the time-dependent UV–Vis absorption spectra of the mixture using a spectrophotometer. Kinetic study of the reaction was also carried out by plotting absorbance (ln A/A_0_) against time (s).

## Results and discussion

### Catalytic characterizations

The nanocatalyst was synthesized following post-grafting approach over the KIT-5 mesoporous silica and Au (0) NP was deposited over the surface functionalized matrix by in situ reduction of the chelated Au(III) ions. The biguanidine moiety was introduced to stabilize the ultrafine Au (0) NPs by electron donation from the densely located N atoms. The as synthesized material was then characterized using different physicochemical techniques like Fourier Transformed Infrared (FT-IR) Spectroscopy, Field Emission Scanning Electron Microscopy (FESEM), Transmission Electron Microscopy (TEM), N_2_-adsorption–desorption analysis, Energy Dispersive X-ray Spectroscopy (EDS) and X-ray diffraction (XRD) study.

**Figure 1:**
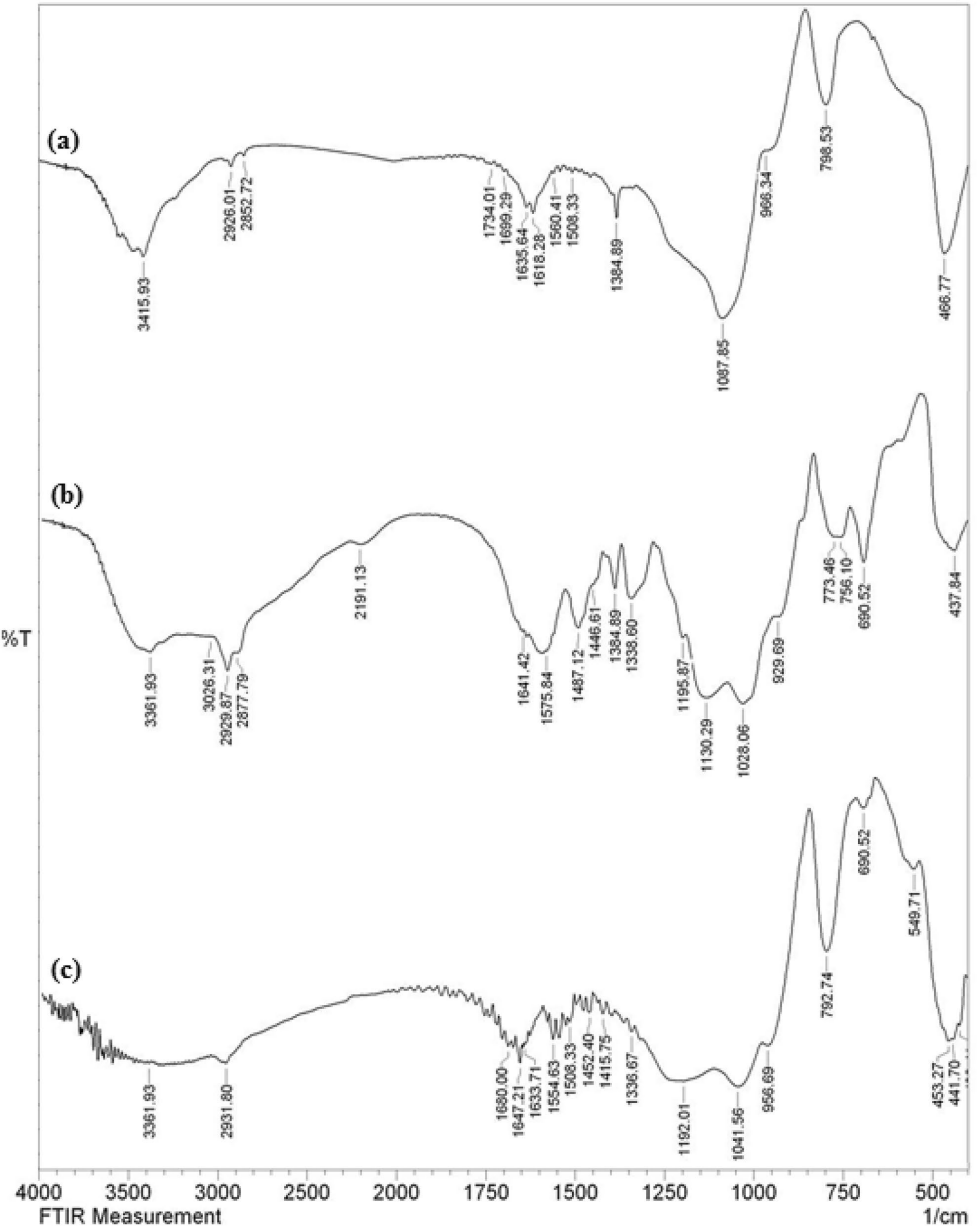
FT-IR spectra of (**a**) KIT-5, (**b**) KIT-5-biguanidine, and (**c**) KIT-5-biguanidine-Au(0).

In order to correlate the stepwise synthesis by organo-ligand functionalization followed by Au (0) deposition over KIT-5, a comparison between the FT-IR spectra of bare KIT-5, KIT-5-NH_2_-guanidine and KIT-5-NH_2_-guanidine-Au(0) materials being recorded in the wavelength region 400–4000 cm^−1^, presented in Fig. 1. In Fig. 1a, the bands appeared at 466, 795 and 1087 cm^−1^ were related to symmetric stretching, symmetric bending and asymmetric stretching vibrations of Si–O–Si bond respectively. The broad peak appeared at 3415 cm^−1^ was due to the silanol groups (Si–OH). Figure 1b, the spectra of KIT-5-biguanidine, depicts some weak peaks at 2877 and 2929 cm^−1^ corresponding to the C–H symmetric and asymmetric stretching frequencies. The broad peak around 3361 cm^−1^ could be the attributed to SiO–H and N–H stretching. The characteristic peaks appeared at 1487 and 1641 cm^−1^ were due to C–N and C=N stretching frequencies from biguanidine function. The presence of these peaks clearly indicate the successful attachment of cyanoguanidine over KIT-5-NH_2_. In Fig. 1c, the stretching frequency due to C=N bond was shifted from 1641 to 1633 cm^−1^ which could be attributed to the coordination Au(0) NPs to biguanidine, implying the immobilization of Au NPs.

**Figure 2:**
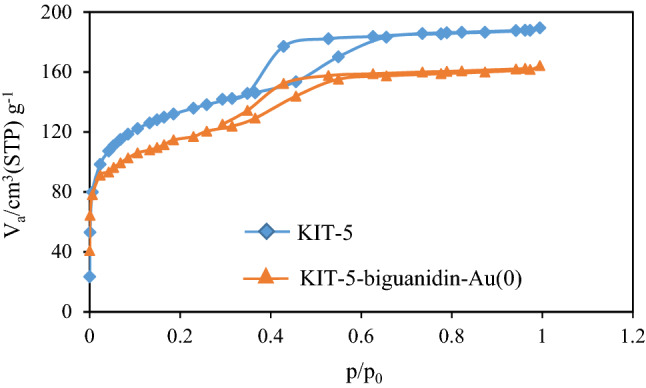
The N_2_ adsorption–desorption pattern of the KIT-5 and KIT-5-biguanidine-Au (0) nanocomposite.

For mesoporous materials, the nitrogen gas adsorption–desorption analysis is an important criteria and the corresponding isotherm has been shown in Fig. 2. Both the bare KIT-5 and Au decorated surface functionalized KIT-5 materials represent a type IV isotherm adjoined with a well-defined H2 hysteresis loop. A sharp capillary condensation occurs at high relative pressure region. In the bare material, the loop closes at 0.4 relative pressure whereas the KIT-5-biguanidine-Au(0) hysteresis loop closes at 0.3, corresponding to lower limit of adsorption. The loop width and height decreases in the substituted functional materials as compared to KIT-5 due to shrink in pore volume and cage size. This also is accompanied by the decrease in surface area. On the other hand, decreasing in the volume pore (Vp), surface area (Sa), and diameter pore (Dp) of KIT-5 can be related to the filling of pores of it by biguanidine and Au nanoparticles. The obtained results from N_2_ adsorption–desorption analysis were presented in Table 1.

**Table 1:** N_2_ adsorption–desorption analysis summarize of KIT-5 and KIT-5-biguanidine-Au (0) samples.

Sample	V_p_ (cm^3^ g^−1^)	S_a_ (m^2^ g^−1^)	D_p_ (nm)
KIT-5	117.14	578.23	5.84
KIT-5-biguanidine-Au(0)	76.12	312.14	3.97

**Figure 3:**
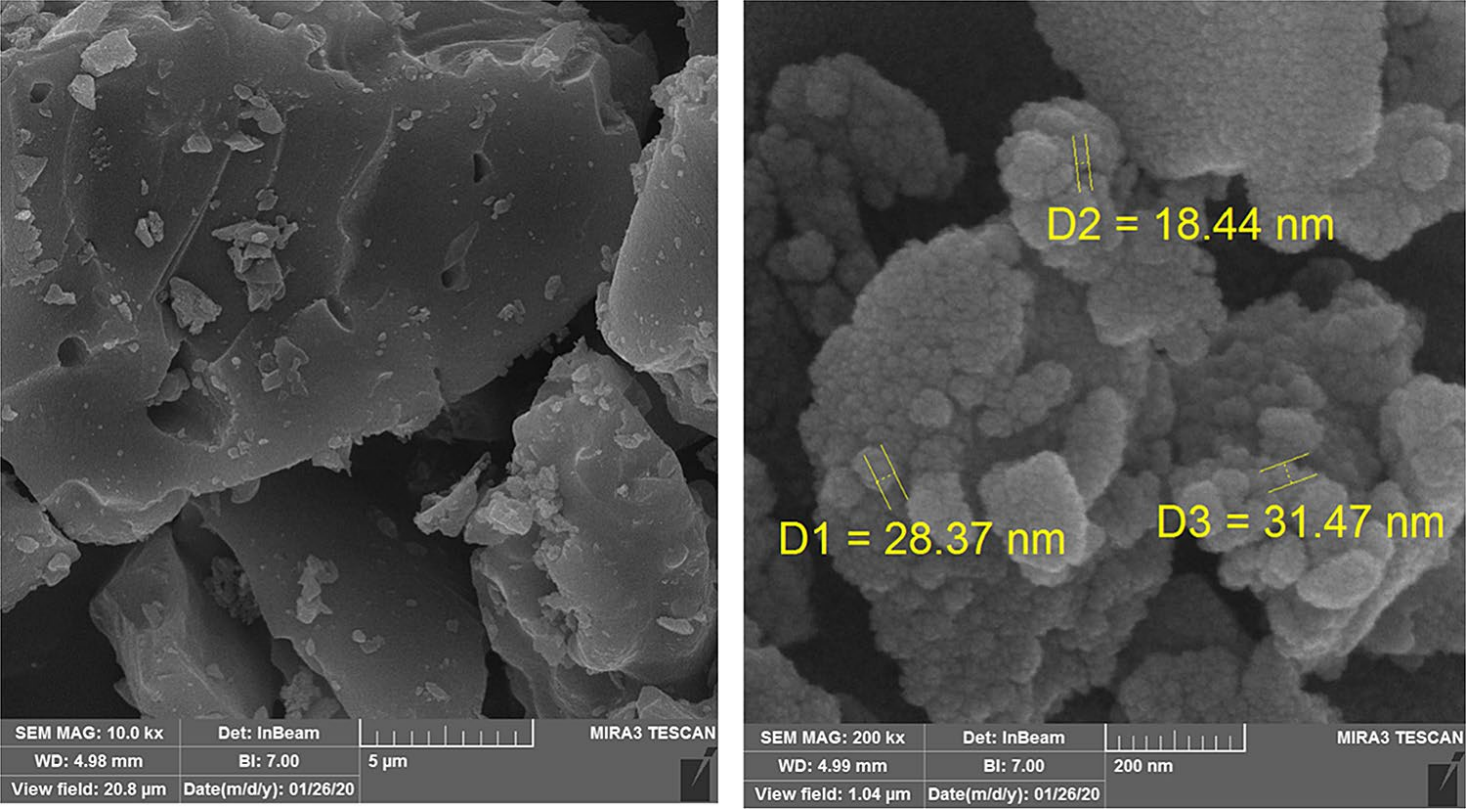
The SEM image of KIT-5-biguanidine-Au (0) nanocomposite.

The structural morphology of the final material has been displayed in Fig. 3 at different magnifications. A closer look of the material represents its floppy and cotton like nature. This is obviously due to its high porosity. Due to higher concentration during sampling the particles seem to be agglomerated. The particle diameter is approximately 29 nm. However, no significant appearance is observed in surface engineered KIT-5 material.

**Figure 4:**
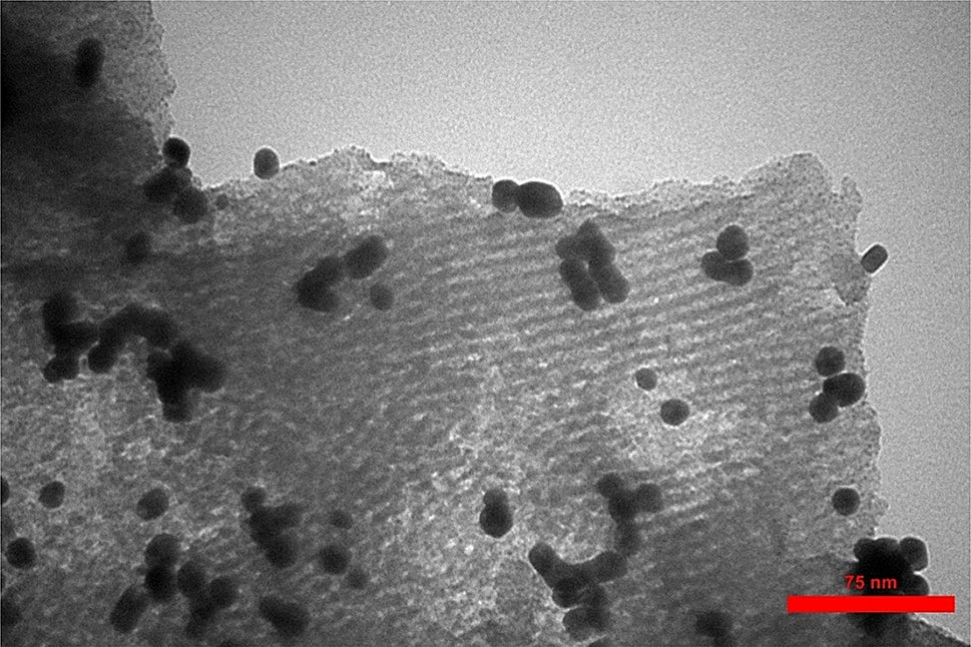
TEM images of the KIT-5-biguanidine-Au(0) nanocomposite.

The more précised intrinsic morphology could be ascertained from TEM images. The 3D ordered cubic mesostructured is presented in Fig. 4. Interconnected porous channels of KIT-5 matrix can be clearly visible from the image. The black dots represent Au NPs, being discretely dispersed throughout the surface of functionalized support. Particle size of the Au NPs were found to be approximately 10–12 nm.

**Figure 5:**
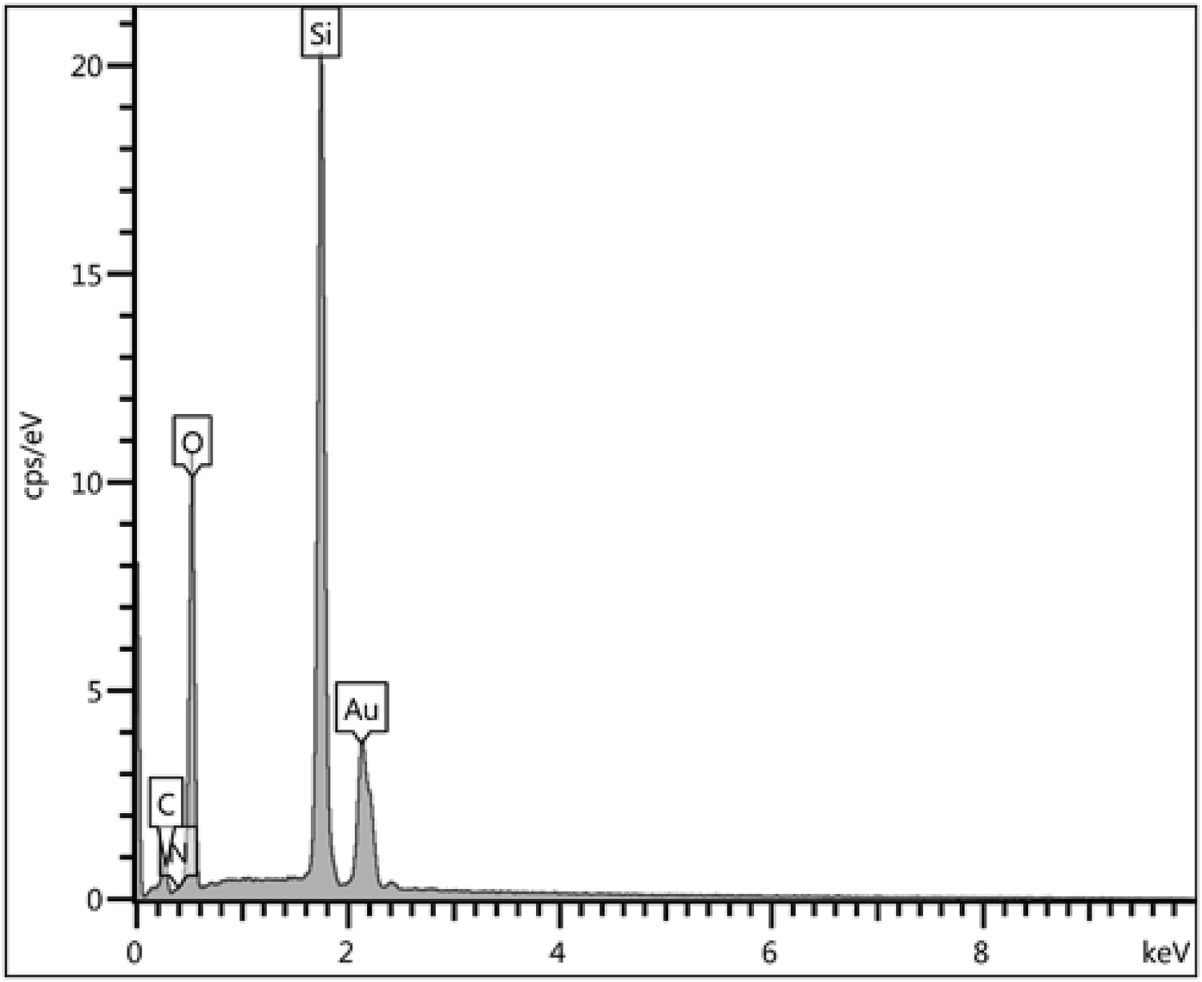
EDX spectrum of the KIT-5-biguanidine-Au (0) nanocomposite.

The EDX analysis was performed to evaluate the purity and possible composition of the synthesized nanocomposite. Figure 5 depicts the spectrum of KIT-5-biguanidine-Au (0) material. It reveals two strong peaks around 2.0 keV due to Si and Au as significant elements. Another sharp peak for O appears around 0.5 keV. Some other small bands corresponding to C and N as elements are also observed in the spectrum. The Si represents KIT-5 material, whereas C, N and O represent the organo functions over KIT-5.

**Figure 6:**
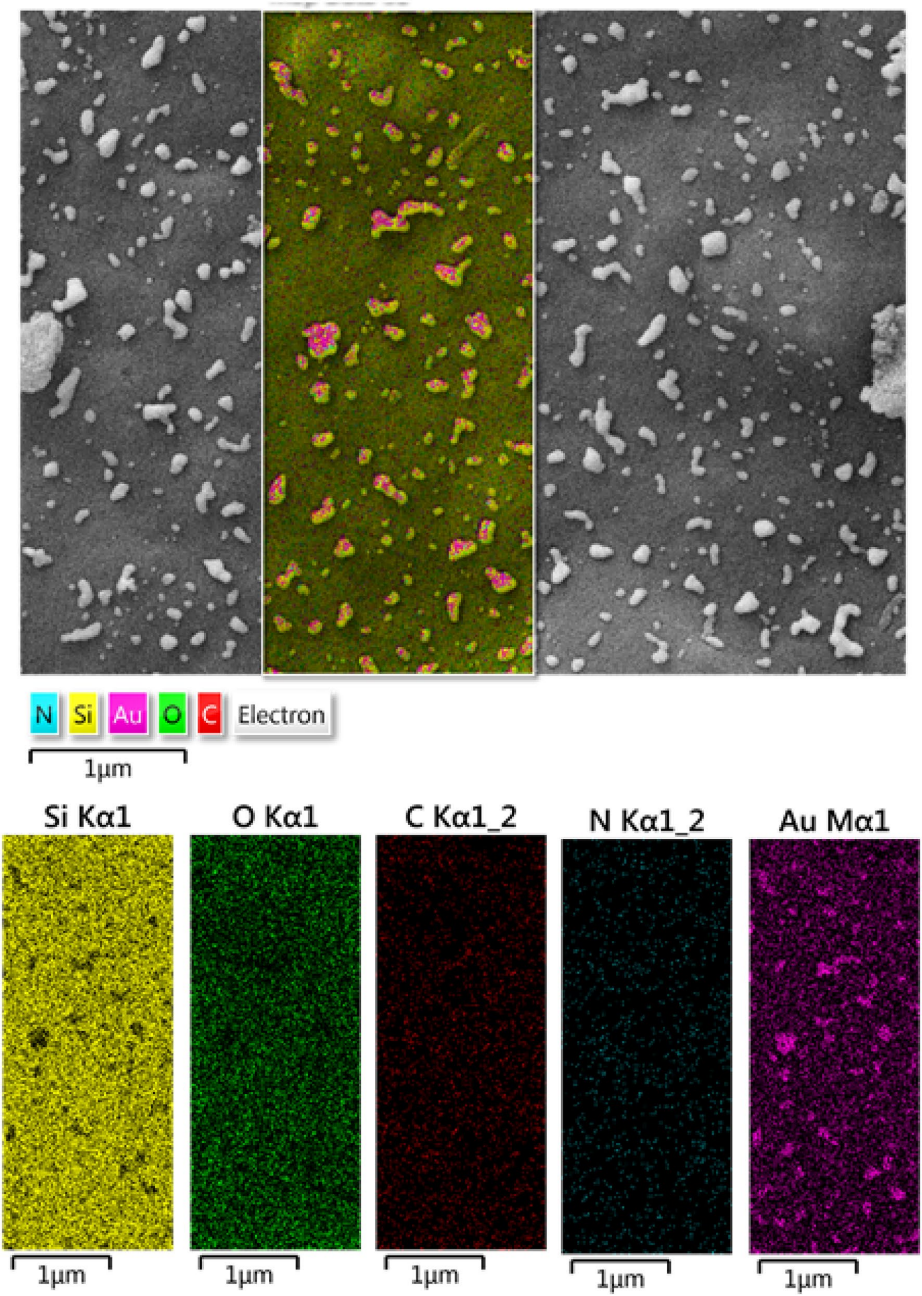
EDS mapping of KIT-5-biguanidine-Au (0) nanocomposite.

On the other hand, EDX elemental mapping analysis of KIT-5-biguanidine-Au (0) nanocomposite was carried out to illustrate the distribution of Si, C, N and Au elements in the composite. Based on Fig. 6, existence of C, N and Au, confirms the successful formation of desired nanocomposite. The figure clearly depicts the homogeneous dispersion of Au NPs on the matrix*.*

**Figure 7:**
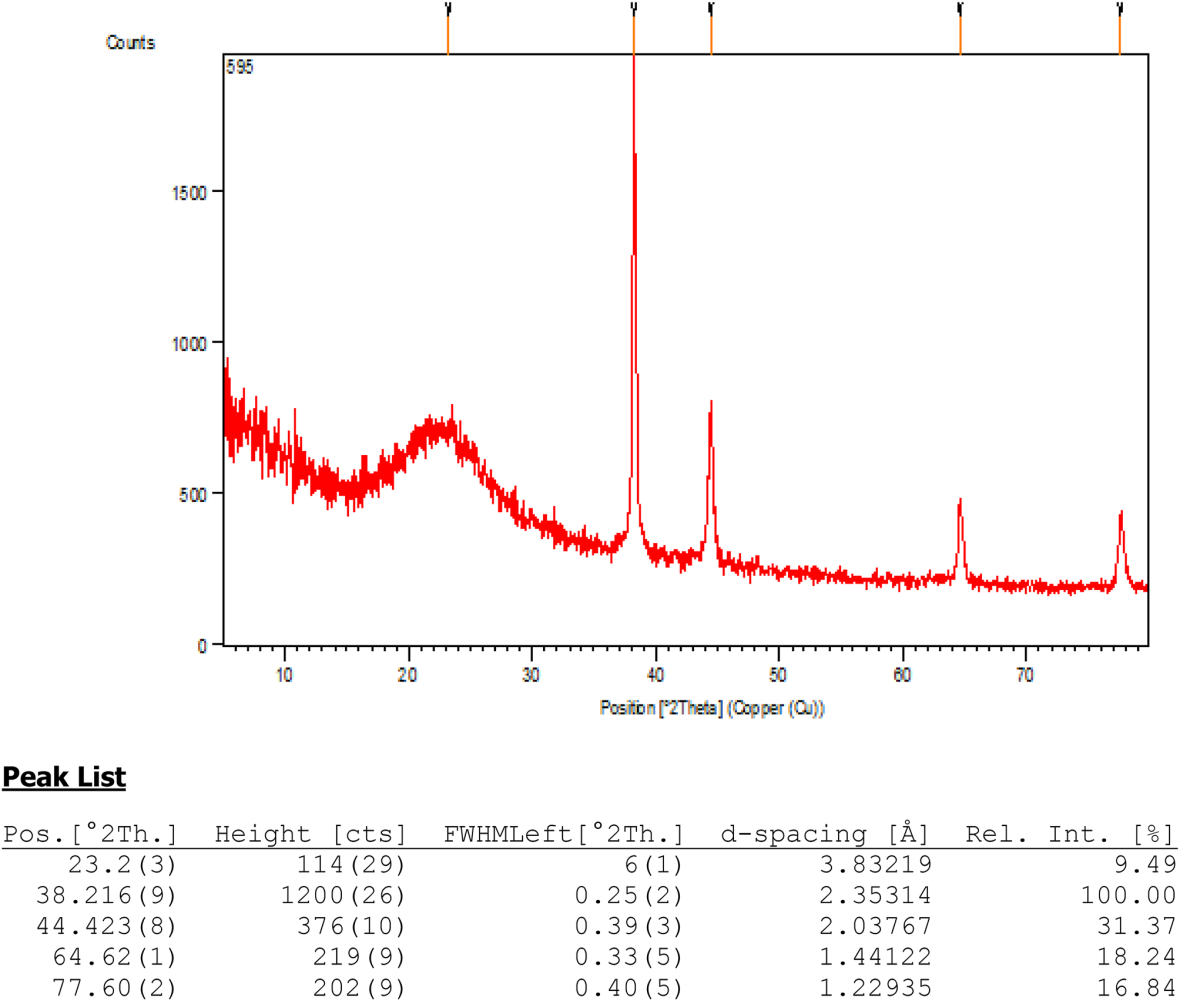
XRD pattern of the KIT-5-biguanidine-Au (0) nanocomposite and its peak list.

Finally, crystalline nature of the KIT-5-biguanidine-Au(0) nanocomposite was validated through the X-ray diffraction analysis. Figure 7, demonstrating the XRD pattern, shows four sharp diffraction peaks and a broad peak. The sharp peaks appeared at 2θ = 38.21°, 44.42°, 64.62° and 77.60° represent the Au face-centred cubic (*fcc*) crystalline phases and corresponds to diffraction on (111), (200), (220) and (311) planes respectively. These characteristic peaks are in close agreement with the standard JCPDS data (File No. 04–0784). The broad peak appeared at 2θ = 23.2° signifies the amorphous silica from KIT-5 base. The oxidation state of Au NPs was investigated by XPS analysis of the nanocomposite in the region of Au 4f. that confirms the presence of Au^0^ species having binding energies of 84.2 eV and 87.8 eV respectively. However, the oxidic species of gold (Au^3+^) is also detected at 82.8 eV and 86.5 eV which might be due to aerial oxidation of Au (0) on exposure during the XPS sampling.

### Catalytic reduction of MB, MO and RhB over KIT-5-biguanidine-Au (0) nanocomposite

**Figure 8:**
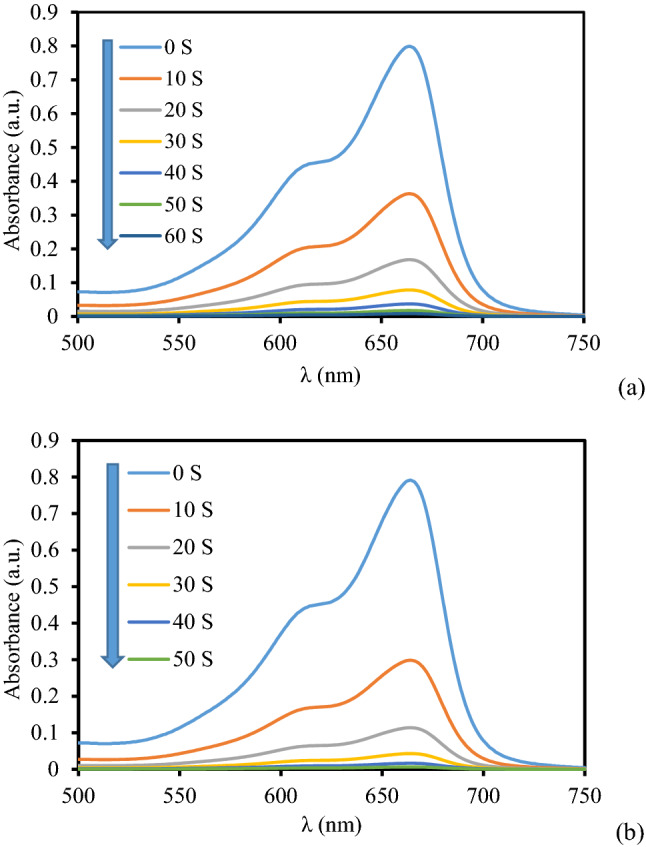
The absorption profile in the reduction of MB using 2 mg (**a**) and 3 mg (**b**) of KIT-5-biguanidine-Au(0) nanocomposite.

**Figure 9:**
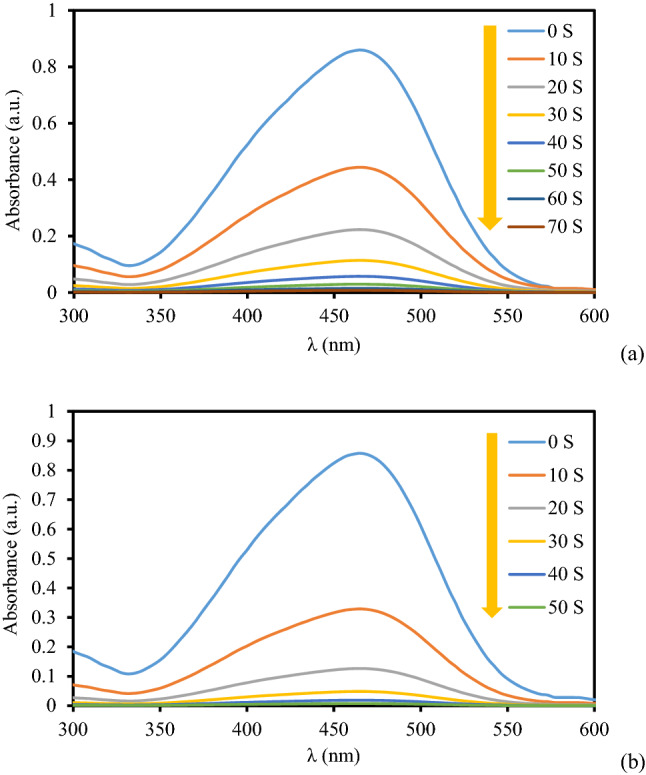
The absorption profile in the reduction of MO using 2 mg (**a**) and 3 mg (**b**) of KIT-5-biguanidine-Au(0) nanocomposite.

**Figure 10:**
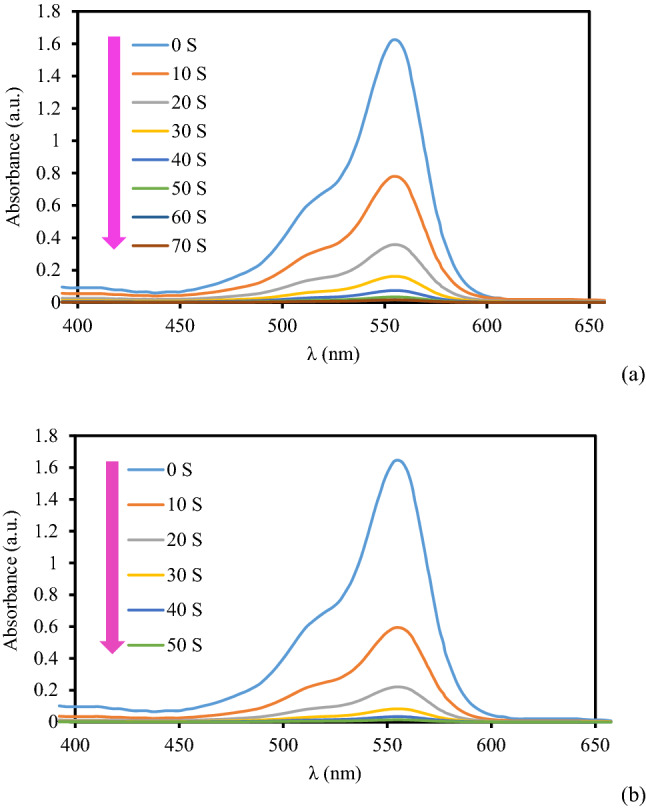
The absorption profile in the reduction of RhB using 3 mg (**a**) and 4 mg (**b**) of KIT-5-biguanidine-Au(0) nanocomposite.

After thorough characterization of our synthesized material, it was the turn to explore its catalytic efficiency. Hence, we focused our attention in investigating the reduction of three water contaminating organic dyes, viz., MB, MO and RhB in presence of NaBH_4_ as reducing agent over our catalyst. The reaction was totally monitored using a UV–Visible spectrophotometer. Dilute aqueous solution of the dye assumed a specific color prior to the addition of borohydride and catalyst. As the reaction started, color of the solution started fading within seconds, an indication of the degradation. This change in color is associated with the change in absorption frequency (*λ*_max_) as recorded by the UV–Vis spectrophotometer. Figures 8,9and10 displays the time dependent absorbance plots of the corresponding dyes. The results corresponding to the reduction of methylene blue in presence of 2 mg and 3 mg of catalyst has been presented in Fig. 8a,b respectively. As seen from the plots, the initial absorption maxima at 660 nm gradually diminishes with time. Interestingly, at higher loading of catalyst the reaction becomes faster and requires smaller time towards complete reduction (60 s vs 50 s), keeping the NaBH_4_ concentration constant. and The similar trend was followed in the reduction of MO (*λ*_max_ 465 nm) and RhB (*λ*_max_ 550 nm) where higher concentration afforded better results towards decoloration of the corresponding dyes and completed in shorter reaction times (70 s vs 50 s for both). The results have been displayed in Figs. 9 and 10 respectively. The more amounts of catalyst were tested and the results showed that by following of increase in the amount of catalyst the rate of reaction increased and reaction time decreased (Table 2). For example, the diagram of the two tests presented. Also, the reduction reaction of organic dyes in the presence of the catalyst and NaBH_4_ alone was tested and no progress observed.

**Table 2:** The kapp of reduction reaction of MB, MO, and RhB with the amount of KIT-5-biguanidine-Au (0) nanocomposite.

Amount of catalyst (mg)	1	2	3	4	5
Kapp (s^−1^), MB	0.057	0.076	0.098	0.109	0.117
Kapp (s^−1^), MO	0.053	0.069	0.099	0.112	0.121
Kapp (s^−1^), RhB	0.046	0.058	0.079	0.097	0.119

**Figure 11:**
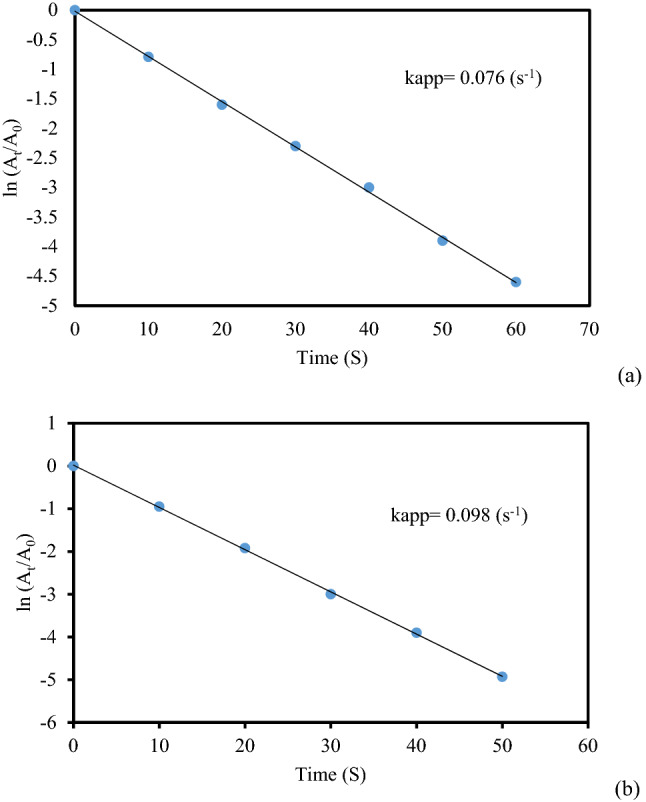
The kinetic plots (lnA_t_/A_0_ vs t) for the reduction of MB using (**a**) 2 mg and (**b**) 3 mg of KIT-5-biguanidine-Au(0) nanocomposite.

**Figure 12:**
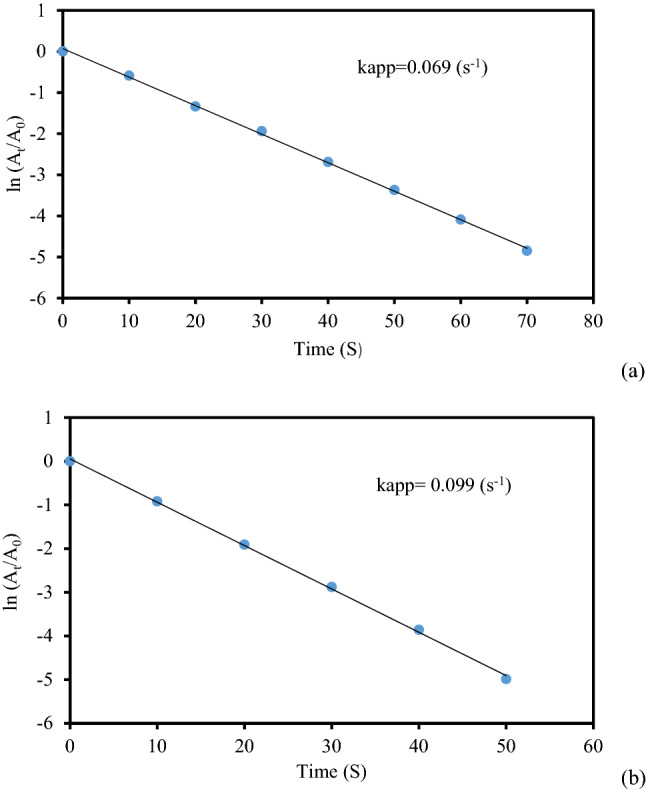
The kinetic plots (lnA_t_/A_0_ vs t) for the reduction of MO using (**a**) 2 mg and (**b**) 3 mg of KIT-5-biguanidine-Au(0) nanocomposite.

**Figure 13:**
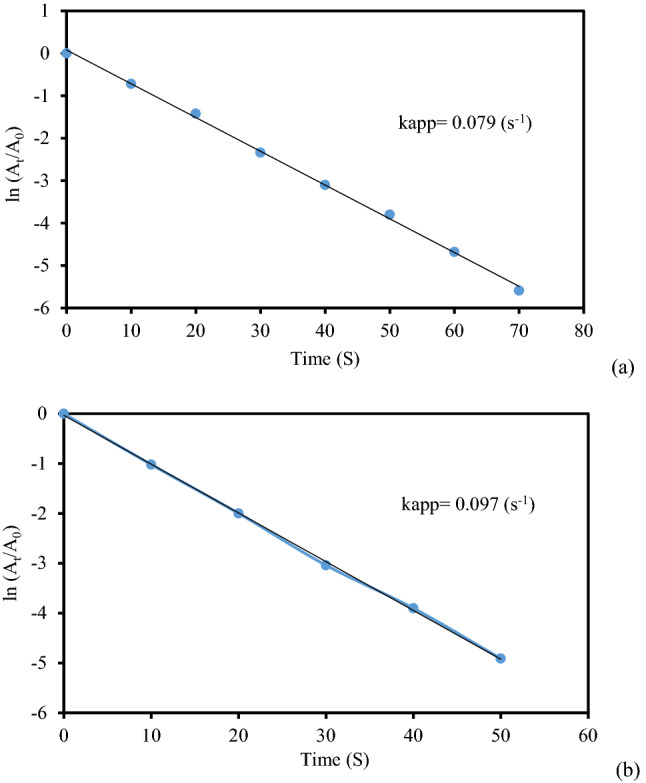
The kinetic plots (lnA_t_/A_0_ vs t) for the reduction of RhB using (**a**) 3 mg and (**b**) 4 mg of KIT-5-biguanidine-Au(0) nanocomposite.

Simultaneously, we also studied the reaction kinetics for the said reactions. As the concentration of NaBH_4_ remained constant throughout the reaction, it can be assumed that the reaction followed pseudo-unimolecular kinetics and accordingly, ln(A_t_/A_0_) = kt, where A_0_ is the initial absorbance and A_t_ is the absorbance at time t in second. On computing, the slope k becomes the apparent reduction rate constant. In so doing, the rate constants for catalytic reduction of MB appeared 0.076 and 0.098 s^−1^ using 2 and 3 mg of catalysts respectively. The plots have been shown in Fig. 11a,b. Similarly, in the catalytic reduction of MO the rate constants were found to be 0.069 and 0.099 s^−1^ whereas for the reduction of RhB those were 0.079 and 0.097 s^−1^ respectively and the corresponding plots have been presented in Figs. 12 and 13.

The control experiments for the reduction of MO, MB and RhB were tested. The results showed that in both conditions without NaBH4 and in the presence of the KIT-5-biguanidine there is no progress in the reduction reaction of MO, MB and RhB after 24 h. Also, the reduction reaction was done in the presence of KIT-5-Pd/NaBH_4_, which showed the end time of the reduction reaction was longer than of the KIT-5-biguanidine-Pd/NaBH_4_ (Table 3).This result showed that the biguanidine can be affected on the catalytic activity of KIT-5-biguanidine-Pd.

**Table 3:** The control experiments for the reduction of MO, MB and RhB.

Catalyst	RhB, yield (time)	MO, yield (time)	MB, yield (time)
KIT-5-Pd/NaBH_4_	99% (180 s)	99% (130 s)	99% (120 s)
KIT-5-biguanidine-Pd/NaBH_4_	99% (70 s)	99% (50 s)	99% (50 s)
KIT-5-biguanidine-Pd	N.R (24 h)	N.R (24 h)	N.R (24 h)
KIT-5-biguanidine	N.R (24 h)	N.R (24 h)	N.R (24 h)

### Mechanism of reduction of dyes by KIT-5-biguanidine-Au (0) nanocatalyst

**Scheme 3:**
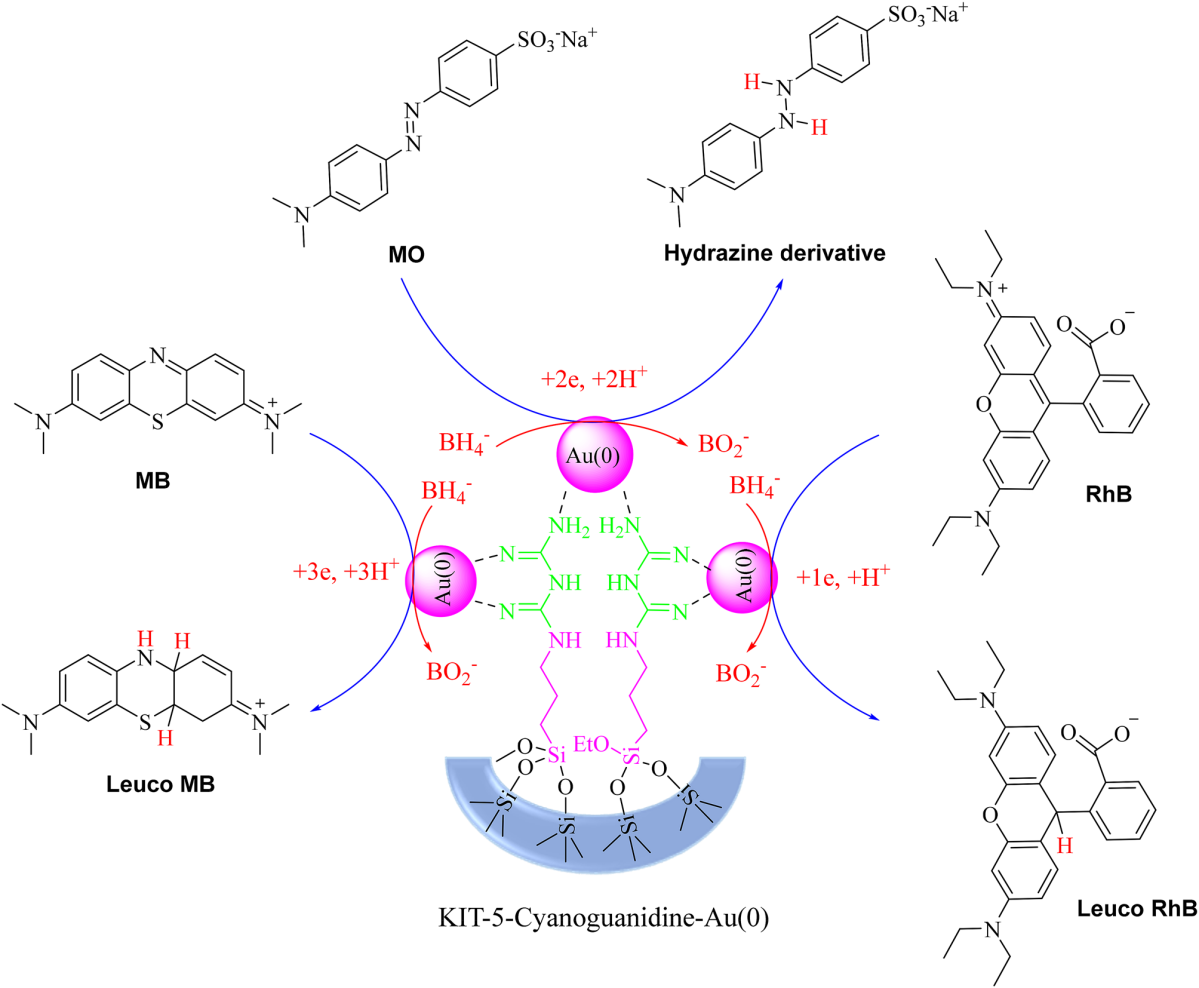
Mechanism of reduction of dyes by KIT-5-biguanidine-Au (0) nanocatalyst.

The reduction of MO, MB and RhB dyes in the presence of KIT-5-biguanidine-Au (0) nanocatalyst and NaBH_4_ is as follows (Scheme 3), in the first step, Au as H^−^ acceptor, and BH_4_^−^ molecules as H^−^ donor are adsorbed on the surface of the nanocatalyst via the π-π interaction, hydrogen bonding and electrostatic interaction. In other words, these molecules (Organic dyes) diffuse from the solution bulk to the surface of nanocatalyst. In the next step, electrons transfer from BH_4_^−^ to the organic dyes, MO, MB or RhB, and this process leads to the reduction and decolorization of dyes (Fig. 9). The formed product desorbs from the surface of the nanocatalyst and diffuses from the surface to the bulk region. The cycle again continues after the evacuation of the active sites by desorption of the products.

### Heterogeneity test and reusability of KIT-5-biguanidine-Au (0) nanocatalyst

Nowadays, one of the important highlights of catalyst is reusability of it, so we were done heterogeneity test and reusability of prepared nanocatalyst. To determine the heterogeneity of the prepared nanocatalyst, the catalyst was separated by a hot filtration test from the reaction mixture after 30 min and was allowed to continue the reaction. For this reaction was not observed a significant increase in the yield of it, can be concluded the catalyst is a heterogenic catalyst. In the other hands, to stability of catalyst was done reusability of it. For this aim the reaction was done in the same reaction under the mentioned conditions in this research.

**Figure 14:**
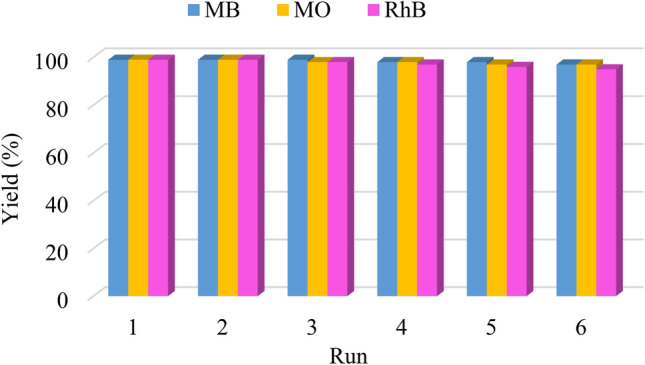
Reusability diagram of KIT-5-biguanidine-Au (0) Nanocatalyst for reduction of MB, MO and RhB.

After reaction the nanocatalyst was collected by centrifugation, washed with ethanol/H_2_O, dried, and reused for the next run. This protocol was done for 6 runs without any significant change in catalytic activity of catalyst (Fig. 14). Also the ICP-AES analysis of the catalyst after 6 runs was shown that amount of Au loaded on the catalyst is 0.30 mmol/g, that was shown a slight change in amount of Au (0.32 to 0.30 mmol/g). It can be concluded that the catalyst has good stability for the reduction of dyes.

## Conclusion

In summary, we have been able to synthesize an Au (0) NP fabricated organ ligand functionalized mesoporous silica nanocomposite [Au(0)-biguanidine-KIT-5]. After proper characterizations the material was explored in the catalytic degradation of several organic dyes like MB, MO and RhB, which have been known to be important contaminants to natural waters and indirectly harmful to human ecology. The catalytic reductions were carried out in presence of NaBH_4_ as reducing agent. The nanocomposite was observed to play its catalytic role tremendously and the colored dyes were faded out within some seconds as monitored in UV–Vis spectrophotometer. This promising catalyst has the potential for being used in large scale applications in the environmental safety programs.
